# Embedding a Choice Experiment in an Online Decision Aid or Tool: Scoping Review

**DOI:** 10.2196/59209

**Published:** 2025-03-21

**Authors:** Nyantara Wickramasekera, Phil Shackley, Donna Rowen

**Affiliations:** 1 Sheffield Centre for Health and Related Research (SCHARR) The University of Sheffield Sheffield United Kingdom

**Keywords:** decision aid, decision tool, discrete choice experiment, conjoint analysis, value clarification, scoping review, choice experiment, database, study, article, data charting, narrative synthesis

## Abstract

**Background:**

Decision aids empower patients to understand how treatment options match their preferences. Choice experiments, a method to clarify values used within decision aids, present patients with hypothetical scenarios to reveal their preferences for treatment characteristics. Given the rise in research embedding choice experiments in decision tools and the emergence of novel developments in embedding methodology, a scoping review is warranted.

**Objective:**

This scoping review examines how choice experiments are embedded into decision tools and how these tools are evaluated, to identify best practices.

**Methods:**

This scoping review followed the PRISMA (Preferred Reporting Items for Systematic Reviews and Meta-Analyses extension for Scoping Reviews) guidelines. Searches were conducted on MEDLINE, PsycInfo, and Web of Science. The methodology, development and evaluation details of decision aids were extracted and summarized using narrative synthesis.

**Results:**

Overall, 33 papers reporting 22 tools were included in the scoping review. These tools were developed for various health conditions, including musculoskeletal (7/22, 32%), oncological (8/22, 36%), and chronic conditions (7/22, 32%). Most decision tools (17/22, 77%) were developed in the United States, with the remaining tools originating in the Netherlands, United Kingdom, Canada, and Australia. The number of publications increased, with 73% (16/22) published since 2015, peaking at 4 publications in 2019. The primary purpose of these tools (20/22, 91%) was to help patients compare or choose treatments. Adaptive conjoint analysis was the most frequently used design type (10/22, 45%), followed by conjoint analysis and discrete choice experiments (DCEs; both 4/22, 18%), modified adaptive conjoint analysis (3/22, 14%), and adaptive best-worst conjoint analysis (1/22, 5%). The number of tasks varied depending on the design (6-12 for DCEs and adaptive conjoint vs 16-20 for conjoint analysis designs). Sawtooth software was commonly used (14/22, 64%) to embed choice tasks. Four proof-of-concept embedding methods were identified: scenario analysis, known preference phenotypes, Bayesian collaborative filtering, and penalized multinomial logit model. After completing the choice tasks patients received tailored information, 73% (16/22) of tools provided attribute importance scores, and 23% (5/22) presented a “best match” treatment ranking. To convey probabilistic attributes, most tools (13/22, 59%) used a combination of approaches, including percentages, natural frequencies, icon arrays, narratives, and videos. The tools were evaluated across diverse study designs (randomized controlled trials, mixed methods, and cohort studies), with sample sizes ranging from 23 to 743 participants. Over 40 different outcomes were included in the evaluations, with the decisional conflict scale being the most frequently used in 6 tools.

**Conclusions:**

This scoping review provides an overview of how choice experiments are embedded into decision tools. It highlights the lack of established best practices for embedding methods, with only 4 proof-of-concept methods identified. Furthermore, the review reveals a lack of consensus on outcome measures, emphasizing the need for standardized outcome selection for future evaluations.

## Introduction

Understanding patient values for treatments is particularly important when a demonstrably superior treatment option is not available [1-4]. In these “preference-sensitive decisions” patients need to understand the treatment choices that are available to them, consider their personal values and weigh the trade-offs between treatment benefits and risks to select the optimal treatment that suits them [1-4]. Patient decision aids are used as a supporting tool when patients are faced with a preference-sensitive decision [5,6]. A well-established evidence base of 209 studies shows the effectiveness of decision aids in improving knowledge, reducing decisional conflict, increasing participation in decision-making, and receiving treatment with characteristics that they value [5,7-9]. Notably, digital decision aids also offer distinct advantages over other formats. Digital tools can incorporate interactive elements, allowing patients to personalize their experience by selecting the most relevant information. Moreover, these tools can include algorithms and perform real-time calculations to provide personalized results [7].

An important part of a decision aid, according to the International Patient Decision Aids Standards (IPDAS) collaboration, is an exercise that helps people clarify their values [10]. A value clarification exercise in a decision aid helps patients to identify the relative importance of treatment characteristics (attributes) that are congruent with their values [10] and helps patients to understand how the different treatment options align with their values [7,11].

Increasingly, choice experiments are used as a value clarification method within decision aids [7,11]. The term choice experiment (also called stated preference survey) will be used hereafter as an umbrella term for discrete choice experiments (DCEs) and conjoint analyses. In choice experiments, patients choose their preferred treatment from two or more hypothetical options, each defined by a unique combination of attributes and levels. Subsequent regression analyses of their choices reveal their preferences for the relative importance of treatment characteristics and allow the prediction of treatments that patients would prefer [12].

The field of embedding choice experiments in decision tools has seen an increase in new research since Weernink et al [7] published their review in 2018. Also, new conceptual models on how to integrate choice experiments in decision tools have been published since 2020 [13,14]. To identify best practices and knowledge gaps considering these advancements, a scoping review is warranted. A scoping review is appropriate as it aims to produce a comprehensive map of the research landscape by analysing the volume of research, the methodologies used, and the overall characteristics of the primary studies [15-20]. Unlike systematic reviews, which mainly focus on establishing the effectiveness of decision aids (already addressed by [5]), scoping reviews map the range of approaches used within a field. This broader approach allows the identification of best practices for integrating choice experiments into online decision aids.

The aim of this study is to undertake a scoping review of choice experiments embedded in decision tools to understand the current landscape of best practices. The objectives of this review are to (1) identify key methods used to embed a choice experiment into a decision tool, (2) characterize the design features of choice experiments embedded in decision aids, (3) identify the different study designs and outcomes used to evaluate the tool, and (4) describe how complex information was presented to the participants.

## Methods

### Overview

This scoping review was conducted following best practices in line with the PRISMA-ScR (Preferred Reporting Items for Systematic Reviews and Meta-Analyses extension for Scoping Reviews) [21]. A protocol detailing the plan of the methods section was prepared and the PRISMA-ScR checklist was used in this review (Multimedia Appendix 1).

### Eligibility Criteria

Studies were selected based on the inclusion and exclusion criteria detailed in Textbox 1.

**Inclusion and exclusion criteria.**

**Inclusion criteria**

- A study describes the methods, development, design, presentation and visualization of patient information of a patient decision tool incorporating a choice experiment.
- A study evaluates a patient decision tool embedding a choice experiment or protocols describing the methods of the evaluation and what outcomes were measured to determine the effectiveness of the intervention.

It should also be noted that

- All study designs such as randomized controlled trials, observational studies, cross-sectional studies, and methodological papers, were included. Conference abstracts and existing systematic reviews were excluded.
- All articles meeting the above eligibility criteria were included regardless of the methodological quality as the aim of the scoping review is to gain an overview of the literature on embedding choice experiments in decision tools.
- No restrictions were imposed on the population, for methodological studies population is not relevant and for evaluation studies, the population can include all users that benefit from the decision tool such as patients, carers, or family members.

**Exclusion criteria**

- Decision aids without a choice experiment task as a value clarification method.
- Decision aids including other preference elicitation methods such as multicriteria decision analysis or time trade-off.
- Non–English-language publications.

### Databases

MEDLINE via Ovid, PsycInfo via Ovid, and Web of Science databases were searched in March 2023. These 3 databases provide sufficient breadth of coverage of health-related sources to identify potentially relevant articles. Reference lists of included studies were searched to identify potentially relevant searches. Internet search engines (ie, Google) were used to identify any gray literature such as unpublished guidance documentation (ie, from regulators).

### Search Strategy

The search strategy was conducted with the help of an information specialist using an iterative discussion process. The key concepts used in the search strategy are “choice experiment,” “decision aid,” “shared decision making,” “value clarification,” “predicted probability,” and synonyms of these key terms. The search excluded other methods such as “multi-criteria decision,” “analytical hierarchy process,” “time trade-off,” or “standard gamble” since the focus of this review is on choice experiments. The search strategy removed any studies not published in English. A time restriction was also introduced because an existing systematic review conducted a search up until 2016 [7]. Therefore, the current search used a modified version of the Weernink et al [7] search strategy and updated it to find new articles published since 2016. The modified version of the search strategy contained new terms such as “decision tool” and “predicted probability.” The full search strategy is included in Multimedia Appendix 2. The gray literature search used the same keywords mentioned above with Boolean operators. The first 20 potentially relevant records were retrieved for screening.

### Screening

All search results were exported to EndNote and any duplicates were removed. All references were screened by one reviewer (NW). However, if the inclusion of a study was unclear, then discussions were held with the wider team to make a final decision (DR and PS). Initially, the references were screened based on title and abstract, followed by a full-text screening of relevant articles based on the inclusion and exclusion criteria. Details about the screening process, including reasons for exclusion were documented in a flow diagram using the PRISMA (Preferred Reporting Items for Systematic Reviews and Meta-Analyses) template.

### Data Charting

Data charting tables were created in Excel to extract data from included studies. The charting tables were developed iteratively and modified as needed after extracting data from two articles. Data charting was conducted independently by one reviewer (NW). The data items included in the Excel charts were: author, date of publication, name of the tool, country, study aim, disease context, sample size, choice experiment embedding methodology or formula used, number of attributes, number of levels, type of attributes, graphs or visual pictographs used, choice experiment design used, type of choice tasks, number of choice tasks, software program used, description of the patient decision tool (ie, receive feedback on attribute importance or receive a “best match” treatment option that aligns with patients desired attributes, received a report), details about the evaluation, outcomes measured, and mean duration of the task. Since multiple study designs were included, not all the fields were relevant for all studies. For example, for studies that report on methodological aspects, the data charts mainly contained information about the methodology or formula, and the rest of the fields were not applicable.

### Collating, Summarizing, and Reporting the Results

Since the aim of the scoping review is to map the literature, the data summarizing used a narrative synthesis of qualitative data with descriptive statistics of quantitative data. Illustrations were used to visualize the results using tables and figures. Where appropriate existing frameworks were used to categorize lists of qualitative data extracted. The Core Outcome Measures in Effectiveness Trials (COMET) taxonomy [22] was used to systematically classify attributes into core areas and domains. This taxonomy is widely used to classify outcomes of trials [23]. This taxonomy’s breadth and granularity, provided by its 5 core areas and 38 outcome domains, made it suitable for classifying the diverse attributes included in this scoping review. Risk of bias assessments were not conducted since the aim of the study was not to test the rigor of the articles, but rather to get an overview of the current literature on embedding choice experiments in decision tools.

## Results

### Overview

**Figure 1:**
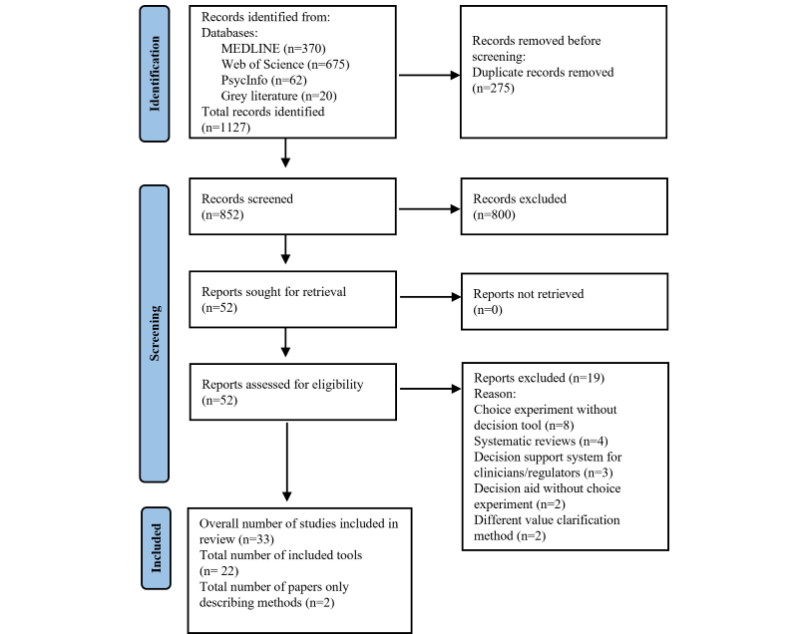
Flow diagram of study selection.

A PRISMA flow diagram detailing the inclusion and exclusion process is presented in Figure 1. A total of 1127 citations were identified. After duplicates were removed, 852 references were screened based on title and abstract. Of the 52 full-text articles that were reviewed, 19 studies that did not meet the inclusion criteria were excluded (Multimedia Appendix 3). Overall, 22 tools were included in the scoping review. The methodology, development and evaluation details of tools were extracted from 33 papers.

### Study Characteristics

Table 1 provides a general overview of the types of decision tools that were found in the scoping review. The first decision tool was published in 2007. However, the number of publications has increased since then, with 16 out of the 22 (73%) studies published since 2015 and a peak of 4 publications in 2019. The majority of the decision tools (17/22, 77%) were developed in the United States. In total, 2 out of 22 (9%) tools were conducted in the Netherlands and one each in the United Kingdom, Canada, and Australia. A variety of different terms such as decision-making tool, decision aid, patient preference elicitation instrument, and discussion prioritization tool were used by the investigators to describe the tool they developed. There is no consensus on a clearly defined name for these tools among the included studies.

**Table 1:** General study characteristics^a^.

Study	Date	Name of the tool	Country	Purpose of tool	Disease context
Abraham et al [24]	2015	Adaptive conjoint analysis survey	United States	Choice of medical treatments - complex antithrombotic therapy (anticoagulants or antiplatelet drugs)	Cardiovascular disease
Almario et al [25]	2018	Online Patient Decision Aid: IBD and me	United States	Choice between biological treatments for ulcerative colitis	Ulcerative colitis and Crohn’s disease
Chhatre et al [26]	2021	Patient preference elicitation instrument: OABCare	United States	Overactive bladder management - relative importance of the key outcomes associated with overactive bladder management (behavioral modification, medications, physical therapy, etc)	Overactive bladder
Cole et al [27]	2022	Electronic health care tool: PRIME	United States	Choice of chemotherapeutic agents for patients with hematologic malignancies - relative importance of outcomes for blood cancer treatments	Hematologic malignancies
de Achaval et al [28]	2012	Adaptive conjoint analysis tool	United States	Choice of different treatment options (“knee replacement surgery” or “pills and physical therapy” versus “unsure”) and relative importance of attributes and therapy or total knee arthroplasty	Knee osteoarthritis
Dowsey et al [29]	2016	Decision aid in total knee arthroplasty	Australia	Choice of whether or not to undergo total knee arthroplasty surgery	End-stage knee osteoarthritis
Fraenkel et al [30]	2007	Computer tool	United States	Choice of different treatment options for knee pain	Knee pain, osteoarthritis
Goodsmith et al [31]	2021	Computerized conjoint analysis	United States	Choice of weight management treatment for overweight individuals with schizophrenia	Overweight individuals with schizophrenia
Hawley et al [32]	2016	Interactive, web-based, breast cancer treatment decision tool	United States	Choice of locoregional treatment (mastectomy or lumpectomy with radiation)	Breast cancer
Hazelwood et al [13,33,34]	2020, 2018, and 2016	Decision aid	Canada	Choice between two treatments	Early rheumatoid arthritis
Hess et al [35]	2015	Preference elicitation tool	United States	Choice of medical, surgical treatment or opt-out for abnormal uterine bleeding	Abnormal uterine bleeding
Hutyra et al [36]	2019	FTASD^b^ decision tool or Preference-Based Decision Aids	United States	Choice between operative or nonoperative treatment for first-time anterior shoulder dislocation	Anterior shoulder dislocations
Jayadevappa et al [37-39]	2015, 2019a, and 2019b	Patient Preferences for Prostate Cancer Care: PreProCare	United States	Choice between treatment options (ie, active surveillance, surgery, radiation) for early-stage prostate cancer	Prostate cancer
Johnson et al [40]	2016	Patient decision aid	United States	Choice of surgery, radiation therapy or active surveillance for prostate cancer	Newly diagnosed prostate cancer (Men)
Loria-Rebolledo et al [41]	2022	Understanding Persistent Pain Decision Aid Tool: UPP^c^ DAT^d^	United Kingdom	Choice of medication for managing persistent pain	Patients living with persistent pain
Pieterse et al [42]	2019	Value Clarification Method - ABEL study	Netherland	Choice of whether or not to undergo short-course preoperative radiotherapy treatment	Newly diagnosed patients with rectal cancer
Pieterse et al [43]	2010	ACA-questionnaire	Netherland	Choice of surgery vs preoperative radiotherapy (PRT) plus surgery for patients with rectal cancer - relative importance of outcomes for rectal cancer	Rectal cancer
Rochon et al [44] and Fraenkel [45]	2014and 2010	Adaptive conjoint analysis decision aid	United States	Choice of different treatment options for knee pain	Knee pain
Snaman et al [46-48]	2019, 2021, and 2021	Decision-making tool: MyPref	United States	Choice of chemotherapy treatments	Adolescent and young adults with high-risk cancer
Streufert et al [49]	2017	Shared decision-making tool	United States	Choice of operative and nonoperative treatment for first-time anterior shoulder dislocation	First-time anterior shoulder dislocation
Studfts et al [50] and Byrne et al [51]	2020and 2019	Brief Education and a Conjoint Valuation Survey	United States	Choice of lung cancer screening test	Individuals at high risk of lung cancer
Wittnik et al [52,53]	2018 and 2016	Customized Care -Discussion Prioritization Tool	United States	To help patients disclose their stressors to their primary care provider	Patients with multiple chronic medical conditions in primary care

^a^Two of the 33 studies were excluded from this table because they focused on method development [14,54].

^b^FTASD: first-time anterior shoulder dislocation.

^c^UPP: Understanding Persistent Pain.

^d^DAT: digital decision aid tool.

The tools were developed in various disease contexts, which can be broadly grouped into three categories: musculoskeletal (7/22, 32%), oncological (8/22, 36%), and chronic conditions (7/22, 32%; Table 1). Musculoskeletal conditions include knee pain, early rheumatoid arthritis, anterior shoulder dislocations, and end-stage knee osteoarthritis; oncological conditions include breast cancer, prostate cancer, lung cancer, rectal cancer, hematologic malignancies, and adolescent and young adults with high-risk cancer; and chronic conditions include cardiovascular disease, overweight individuals with schizophrenia, ulcerative colitis, and Crohn disease, patients with multiple chronic medical conditions in primary care, and overactive bladder. The majority of the tools (20/22, 91%) were developed to help patients choose between medical treatments or compare medical treatments with surgery or surveillance. However, 2 out of 22 (9%) tools had different purposes, one study was developed to help patients disclose their stressors (ie, mobility issues and money worries) to their primary care provider and the other to facilitate the decision-making process around the choice of lung cancer screening tests.

### Attributes

The various types of attributes that were used to assess patient preferences are listed in Table 2. The COMET taxonomy was used to organize the 91 attributes into meaningful domains and gain insight into the breadth of attributes that were included in the choice tasks. Five core areas and 11 outcome domains were identified when attributes were classified (Table 2). Attributes that were predominantly used in decision tools are: efficacy (20/22, 91%), side effects (15/22, 68%), route of administration (9/22, 41%), cost (7/22, 32%), and limits on daily activities (6/22, 27%). Further details of the attributes are available in Multimedia Appendix 4.

**Figure 2:**
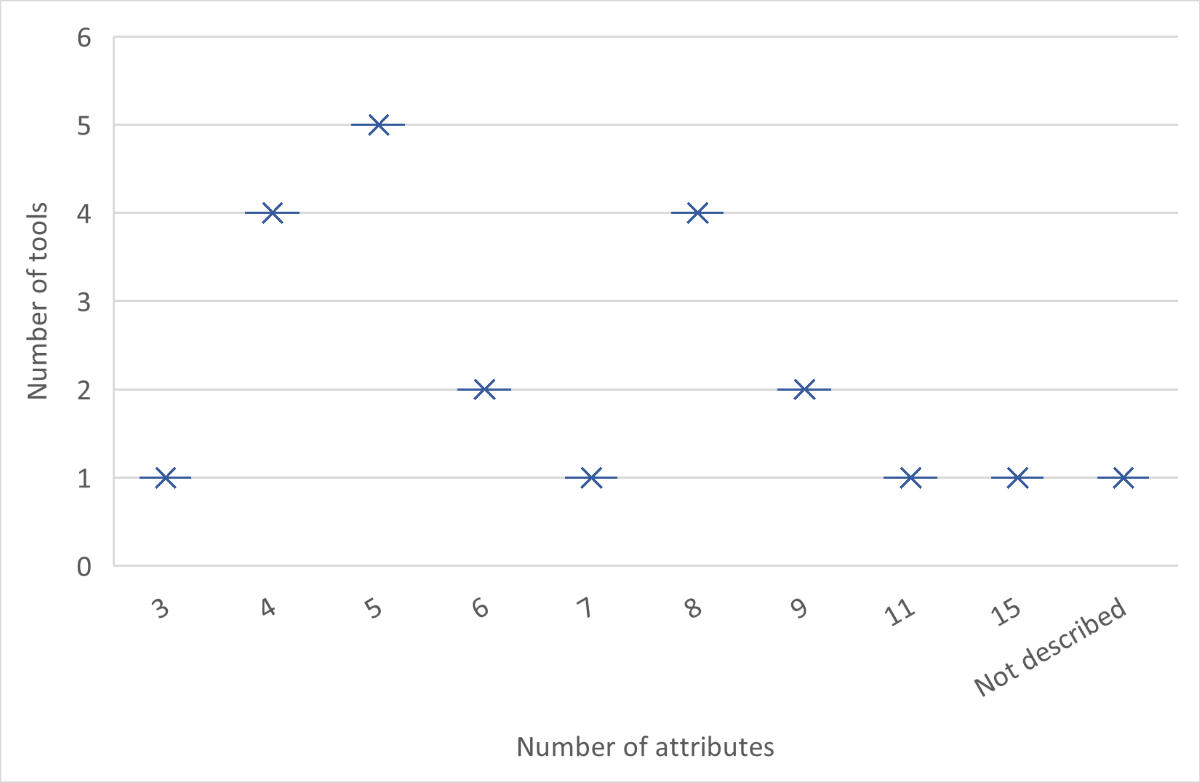
Number of attributes included in the tools.

Overall, a range of attributes from 3 to 15 were used in the decision tools (Figure 2). Most tools (8/22, 36%) with a higher number of attributes (8 or above), used a design that allowed them to simplify the choice task and only display a reduced number of attributes (ie, 3 attributes at a time) to the respondents. Further details about the choice of tasks can be found in Multimedia Appendices 4 and 5.

**Table 2:** Classification of attributes.

Core area, outcome domain, and attributes (in order)				Frequency (%)^a^
**Death**				
	**Mortality or survival**			
		Survival	2 (9)	
**Physiological or clinical**				
	**Physiological or clinical**			
		Efficacy	20 (91)	
		Recurrence	2 (9)	
		How the medication works	1 (5)	
**Life impact**				
	**Functioning**			
		Limits on daily activities	6 (27)	
		Mobility	1 (5)	
		Impact on social life	1 (5)	
		Appearance	1 (5)	
	**Global quality of life**			
		Quality of life	1 (5)	
	**Perceived health status**			
		Stress	1 (5)	
	**Delivery of care**			
		Route of administration	9 (41)	
		Recovery time	3 (14)	
		Treatment location	4 (18)	
		Duration of treatment	2 (9)	
		Need for additional treatment	1 (5)	
		Check-ups	1 (5)	
		Type of provider	1 (5)	
		Delivery mode (Internet or in-person)	1 (5)	
		Frequency of clinic visits	1 (5)	
		Individual or group intervention	1 (5)	
	**Personal circumstances**			
		Safety	1 (5)	
		Food	1 (5)	
		Money worries	1 (5)	
**Resource use**				
	**Economic**			
		Cost	7 (32)	
	**Hospital**			
		Time in hospital	3 (14)	
		Permanence	1 (5)	
	**Societal or care burden**			
		Caregiver burden	1 (5)	
**Adverse events**				
	**Adverse events**			
		Side effects	15 (68)	
		False Positive	1 (5)	

^a^The percentage is calculated by dividing the frequency by the total number of tools (n=22).

**Figure 3:**
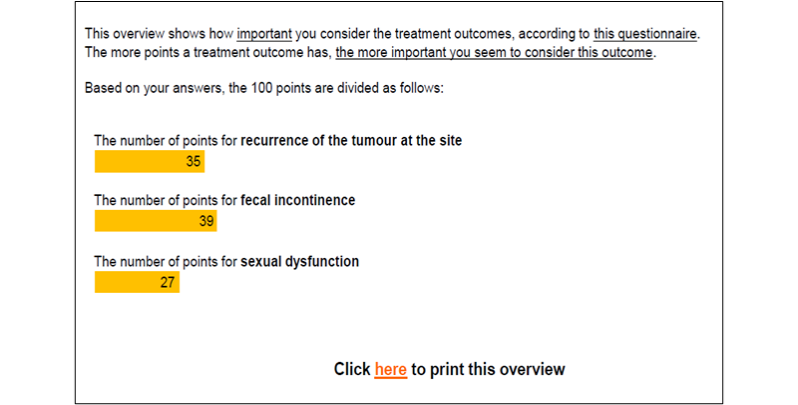
Horizontal bar graph reproduced from [42] which is published under Creative Commons Attribution 4.0 International License [55].

**Figure 4:**
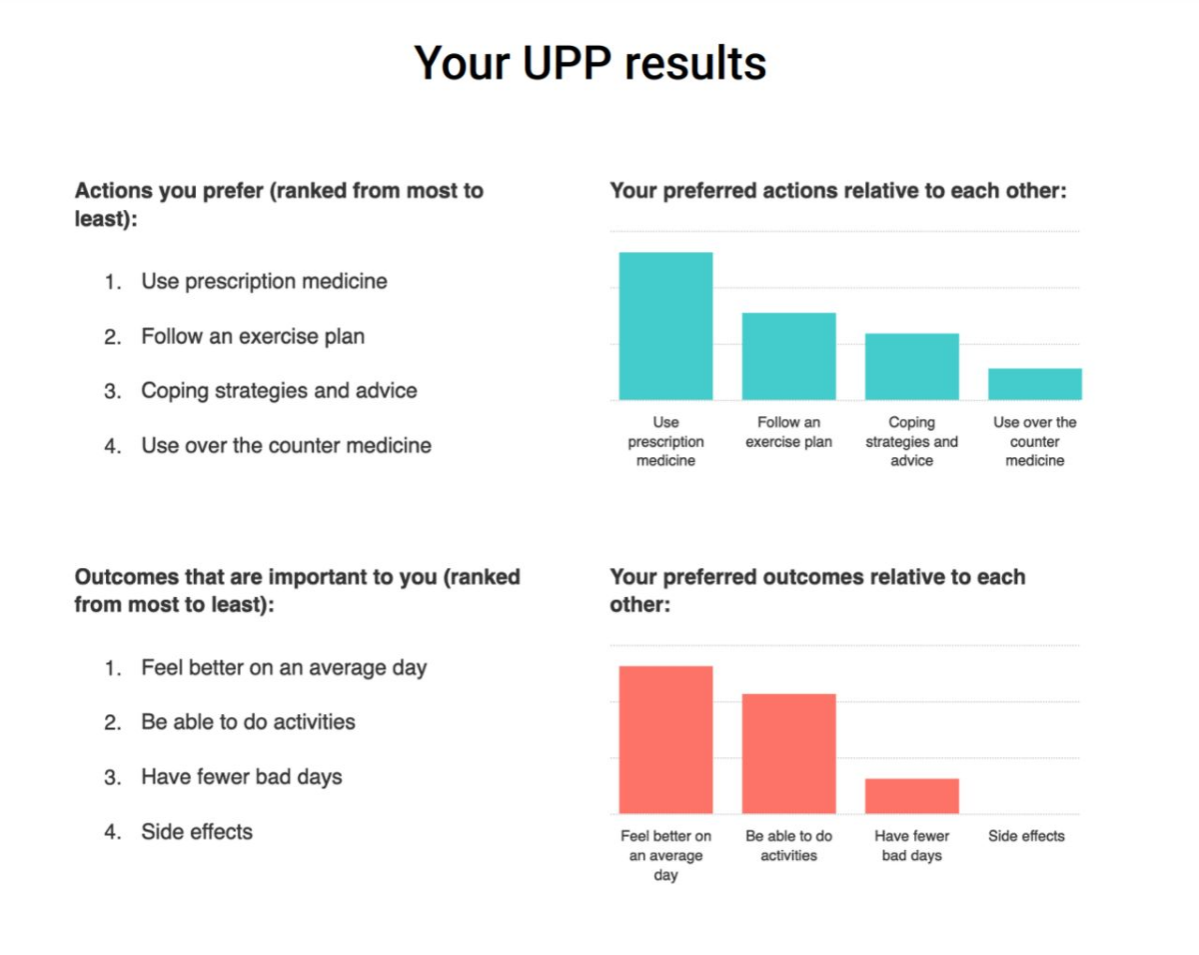
Vertical bar graph reproduced from [41] which is published under Creative Commons Attribution 4.0 International License [56].

**Figure 5:**
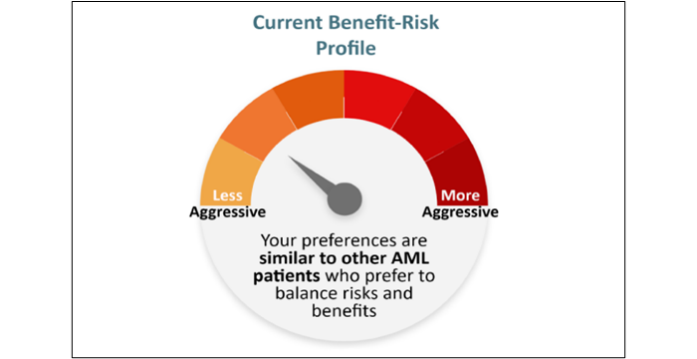
Gauge chart reproduced from [27] which is published under Creative Commons Attribution 4.0 International License [57]. AML: acute myeloid leukemia.

**Figure 6:**
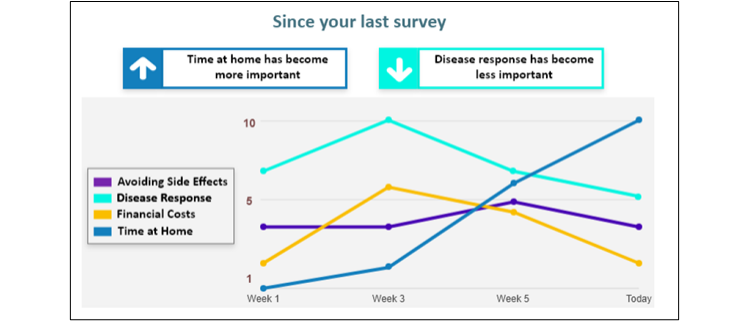
Line graph reproduced from [27] which is published under Creative Commons Attribution 4.0 International License [57].

**Figure 7:**
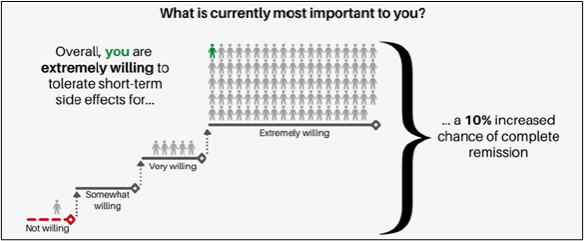
Narrative visualization reproduced from [27] which is published under Creative Commons Attribution 4.0 International License [57].

### Defining Risk or Efficacy Attributes

The majority of tools (20/22, 91%) incorporated probabilistic attributes to convey uncertainty in treatment efficacy and side effects (Table 3). To enhance comprehension, various approaches were used to express these probabilities, including percentages (9/22, 41%), natural frequencies (9/22, 41%), icon arrays (8/22, 36%), narratives (5/22, 23%), and videos (1/22, 5%). Over half of the tools (13/22, 59%) used a combination of methods, while a minority (4/22, 18%) relied on a single approach. In total 14% (3/22) tools did not clearly describe the method used. The most frequent combination was natural frequencies and icon arrays (7/22, 32%).

**Table 3:** Methods used to define the risk or efficacy attributes^a^.

Study	Percentages (ie, 5% risk of the cancer coming back)	Natural frequencies (ie, 10 out of 100 people will have a heart attack)	Icon arrays (numerical data visualized using simple icons like faces)	Narratives (ie, More than half; side effects - likely; low likelihood)	Videos
Abraham et al [24]		✓	✓		
Almario et al [25]	✓	✓	✓		✓
Chhatre et al [26]	✓			✓	
Cole et al [27]	✓			✓	
De Achaval et al [28]		✓	✓		
Dowsey et al [29]		✓	✓		
Fraenkel et al [30]		✓	✓		
Goodsmith et al [31]	N/A^b^	N/A	N/A	N/A	N/A
Hawley et al [32]	✓			✓	
Hazelwood et al [13,33,34]		✓	✓		
Hess et al [35]	Not described	Not described	Not described	Not described	Not described
Hutyra et al [36]	Not described	Not described	Not described	Not described	Not described
Jayadevappa et al [37-39]	✓			✓	
Johnson et al [40]	Not described	Not described	Not described	Not described	Not described
Loria-Rebolledo et al [41]				✓	
Pieterse et al [42]		✓	✓		
Pieterse et al [43]		✓	✓		
Rochon et al [44] and Fraenkel et al [45]	✓	✓			
Snaman et al [46-48]	✓				
Streufert et al [49]	✓				
Studfts et al [50] and Byrne et al [51]	✓				
Wittnik et al [52,53]	N/A	N/A	N/A	N/A	N/A
Total count	9	9	8	5	1

^a^Two of the 33 studies were excluded from this table because they focused on method development [14,54].

^b^N/A: Not applicable.

### Choice Task Design

Five types of designs were used to derive the choice tasks (Table 4). The most frequently used design type was adaptive conjoint analysis (10/22, 45%), followed by conjoint analysis (4/22, 18%), DCEs (4/22, 18%), modified adaptive conjoint analysis (3/22, 14%) and adaptive best-worst conjoint analysis (1/22, 5%). Common characteristics of the choice tasks were the rating of preferred treatments (ie, “strongly prefer left,” to “strongly prefer right”) in 17 out of 22 (77%) tools; and tailoring of choice tasks using various adaptive designs (14/22, 64%). Further details about the different designs can be found in Multimedia Appendix 5.

The number of choice tasks included varied from 6 to 20 (Multimedia Appendix 5). Eight out of 22 tools (36%) did not specify the number of tasks included in the tool. Conjoint analysis typically used a higher range of 16-20 tasks, whereas DCEs and adaptive conjoint analysis designs used a lower range of 6-12 tasks (Multimedia Appendix 5).

**Table 4:** Types of choice of tasks.

Choice task type	Frequency	Details of the choice task
Adaptive conjoint analysis (adaptive choice-based conjoint, choice-based adaptive conjoint analysis)	10	First, participants were shown an attribute with two different levels and asked to rate how important was the level difference (ie, “strongly prefer left or right”). Second, respondents were shown tailored paired comparisons and asked to rate their preferences (ie “strongly prefer left or right”).
Conjoint analysis	4	Paired comparison (or single profile) where respondents rated their preference (ie, strongly prefer treatment option 1/2).
Discrete choice experiment	4	Paired comparisons of treatment profiles where respondents chose one treatment over the other (no rating involved). Efficient designs or balanced overlap designs were generated using Ngene or Sawtooth.
Modified adaptive conjoint analysis	3	First, respondents chose the attribute that is most important to them out of a list of attributes. Second, the respondents rated the importance of each attribute relative to the most important attribute; followed by paired comparisons of treatment profiles which were rated (ie, “strongly prefer left or right”).
Adaptive best-worst conjoint analysis	1	Presented three tailored alternative profiles and asked respondents to choose the best and worst of alternatives.

### Embedding Choice Tasks in Decision Tools

Table 5 details the choice experiment embedding methods used by the 22 decision tools included in the scoping review. In addition, Table 5 presents a summary of the two methodological papers included [14,54]. Fourteen decision tools used Sawtooth software; one study each used Wisercare [40], Dynamic Computer Interactive Decision Application [13] and Clinvivo software [41]; and 5 tools failed to describe the choice task embedding method.

Sawtooth Software dominated, comprising approximately 64% (14/22) of the decision tools, which allowed real-time data analysis and display of personalized results to respondents. However, details on how the Sawtooth software provided real-time tailored results were limited (n=7). Even when the authors provided information beyond the Sawtooth software name, they only mentioned the name of the regression model used such as hierarchical Bayes (n=3) or least-squares regression analysis (n=4) without providing any specific details regarding the model configuration or the mechanisms enabling real-time personalization (Table 5). Similarly, one study used the Wisercare software, but no further details were provided. The remaining four studies used distinct methods to embed choice tasks in decision tools and these methods are summarized in detail below.

Hazelwood et al [13] used the Dynamic Computer Interactive Decision Application templating tool to develop the decision aid [13]. To provide tailored treatment ranking, DCE data from a previous study [34] was analyzed using a Bayesian model followed by scenario analyses [33]. The decision aid contained 6 DCE tasks. By analyzing the results of the prior DCE, a choice probability was calculated for each of the 64 (2^6^) response profiles (ie, AABAAB, ABABAB, BBBAAA…etc) for selecting one of two possible treatments (triple therapy or methotrexate). When a patient completed the 6 choice tasks, the patient was assigned to 1 out of 64 response profiles and the corresponding probability for that response profile of choosing triple therapy or methotrexate was displayed [13].

Gonzalez Sepulveda et al [14] also used data from a prior conjoint analysis [36] to develop a preference diagnostic tool. This paper detailed only the methodology with a simulation study to test the proposed method. First, they analyzed the conjoint analysis data to evaluate the distribution of preferences and identify groups or classes of respondents with similar preferences. The authors refer to these groups as “known preference phenotypes.” To identify preference phenotypes, Gonzalez Sepulveda et al [14] suggested using methods such as k-means cluster analysis, latent class analysis, or hierarchical cluster analysis. The second step involved constructing a small number of choice sets that would “maximize discrimination” of respondents belonging to each preference phenotype. To generate choice sets the authors used an evolutionary algorithm. Third, Gonzales Sepulveda et al [14] evaluated the robustness (ie, true positives or true negatives) of the choice sets on correctly predicting preference phenotypes [14]. In the simulation tasks, the investigators constructed three choice tasks, where all respondents saw choice task 1 and based on the answer to the first choice task, respondents either saw choice task 2 or choice task 3. Each new question classified the respondent to the preference phenotype they were likely to belong to. Based on the response patterns to the two questions, the investigators assigned respondents to their likely preference phenotypes [14]. Gonzalez Sepulveda et al [14] argued that a key advantage of their method is the use of a small number of choice tasks to accurately predict the posterior probability of preference phenotype membership.

**Table 5:** Methods of tool development.

Study	Choice task embedding approach
Abraham et al [24]	Sawtooth software (regression analysis)
Almario et al [25]	Sawtooth software (hierarchical Bayes regression)
Chhatre et al [26]	Sawtooth software (hierarchical Bayesian random effects regression)
Cole et al [27]	Not specified but this tool is a work in progress only protocol published
de Achaval et al [28]	Sawtooth software (no further details provided)
Dowsey et al [29]	Not described
Fraenkel et al [30]	Sawtooth software (least-squares regression analysis)
Gonzalez et al [14]	Developed an algorithm “based on previous information on the distribution of patient preferences in a population” using hierarchical cluster analysis
Goodsmith et al [31]	Not described
Hawley et al [32]	Not described
Hazelwood et al [13,33,34]	Hierarchical Bayes regression from a prior discrete choice experiments (DCEs) is used to find expected choice probabilities of treatments for response patterns. Software used was Dynamic Computer Interactive Decision Application (DCIDA) tool
Hess et al [35]	Sawtooth software (no further details provided)
Hutyra et al [36]	Sawtooth software (no further details provided)
Jayadevappa et al [37-39]	Sawtooth software (no further details provided)
Johnson et al [40]	Wisercare no further details provided)
Loria-Rebolledo et al [41]	Real time estimates using the penalized logit regression coded using Clinvivo software
Pieterse et al [42]	Sawtooth software (Ordinary least squares regression)
Pieterse et al [43]	Sawtooth software (Ordinary least squares regression)
Rochon et al [44] and Fraenkel et al [45]	Sawtooth software (Least squares updating algorithm)
Snaman et al [46-48]	Sawtooth software (no further details provided)
Shaoibi et al [54]	Bayesian collaborative filtering model
Streufert et al [49]	Sawtooth software (no further details provided)
Studfts et al [50] and Byrne et al [51]	Sawtooth software (hierarchical Bayes approach)
Wittnik et al [52,53]	Not described

Shaoibi et al [54] used a Bayesian collaborative filtering model to predict the treatment recommendations. The first step of the proposed method is similar to Gonzales Sepulveda et al [14] as it involves the use of preference phenotypes. A Markov Chain Monte Carlo algorithm was used to identify preference clusters [54]. Then the investigators used data from existing patients to assess posttreatment satisfaction. When a new patient completed the choice task, they were assigned to a preference cluster. Then post treatment satisfaction data that match the cluster were used to make a treatment recommendation.

Loria-Rebolledgo et al [41] used a different approach to the other methods discussed above. This method did not require a 2-stage approach of analyzing data from a previous choice experiment instead, parameters were estimated “live” using a penalized multinomial logit model (pMNL). The Clinvivo software was used to code the decision aid tool. Loria-Rebolledgo et al [41] selected the penalized model because of its flexibility in converging results especially when small sample sizes are used for the estimation. The pMNL is different from a typical multinomial logit model because a bias term is added to the standard likelihood function. This term penalized the model for small sample size bias. Each time a respondent completed the choice task, the pMNL model was run and relative importance scores were calculated.

### Presentation of the Decision Tool

**Figure 8:**
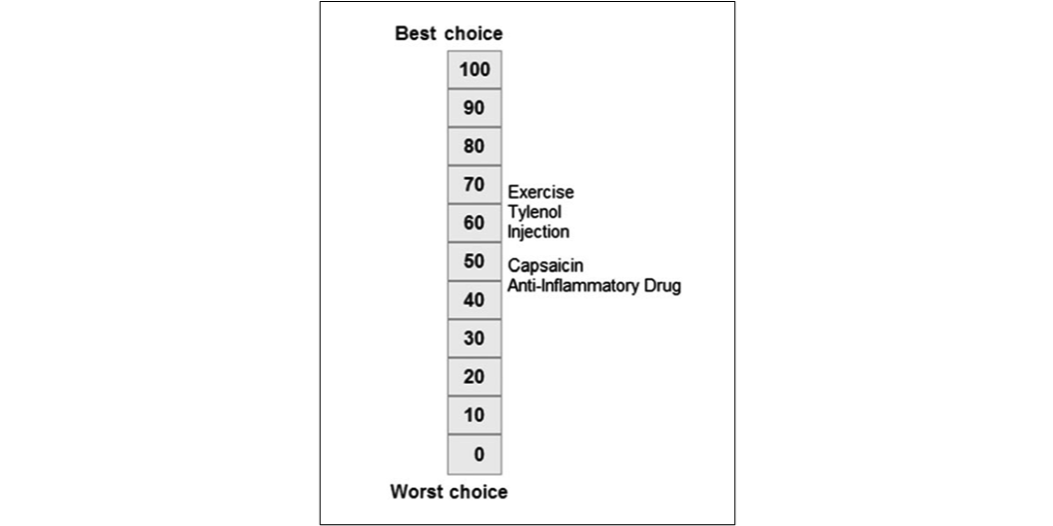
Scale displaying relative ranking of treatments reproduced from [44]. Used with permission of John Wiley and Sons - Books, from Rochon et al, 2014;17(6):840-5; permission conveyed through Copyright Clearance Center, Inc.

**Figure 9:**
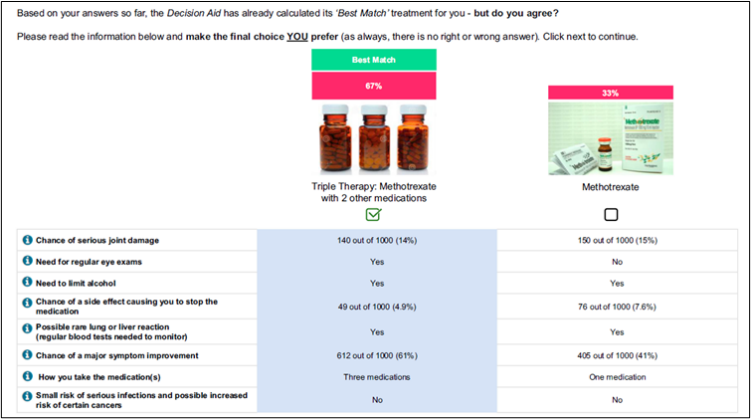
“Best match” treatment presented in a choice task format [13]. Patient Preference and Adherence 2020:14 829-838 - Originally published by and used with permission from Dove Medical Press Ltd.

After completing the choice task, 64% of tools (14/22) provided respondents with a report, while 8 out of 22 (36%) tools did not provide a clear description of what respondents received. Two types of information were included in this report: (1) attribute importance scores and (2) “best match” treatment options that aligned with patients’ desired attributes. Most patients received feedback on attribute importance (16/22, 73%), which was presented to patients using different formats. The majority (14/22, 64%) were displayed as horizontal bar graphs (Figure 3 [42]). There were slight variations in the presentation of horizontal bar graphs: some showed exact percentages, some showed longer bars to represent increasing importance, and some tools with a long list of attributes only presented the top 5 most important attributes. Diverging from horizontal bar graphs, one tool used a vertical bar graph (Figure 4 [41]). Another study tested multiple different approaches such as a gauge chart (Figure 5 [27]), line graph (Figure 6 [27]) and narrative visualization (Figure 7 [27]). Only 5 out of the 22 (23%) tools provided patients with a “best match” treatment. Two tools used a scale ranging from 0 (worst choice) to 100 (best choice) showing the relative ranking of all the available treatment options (Figure 8 [44]). One tool displayed all available treatments in a choice task format and highlighted the “best match” (Figure 9 [13]); one tool picked the treatment that best fits the respondent and only presented that option in the text and the last tool did not provide sufficient details on how the ranking of treatment options was presented to the patients (Multimedia Appendix 6).

### Evaluation of Decision Tools

The decision tools were evaluated in the context of different study designs, including randomized controlled trials (RCTs; n=5), pilot RCTs (n=4), mixed methods studies (n=4), cohort studies (n=4), cross-sectional studies (n=3), qualitative studies (n=1), and nonrandomized studies (n=1). Sample sizes across the studies varied, ranging from 23 participants in mixed methods studies to 743 in RCTs (Multimedia Appendix 7).

**Figure 10:**
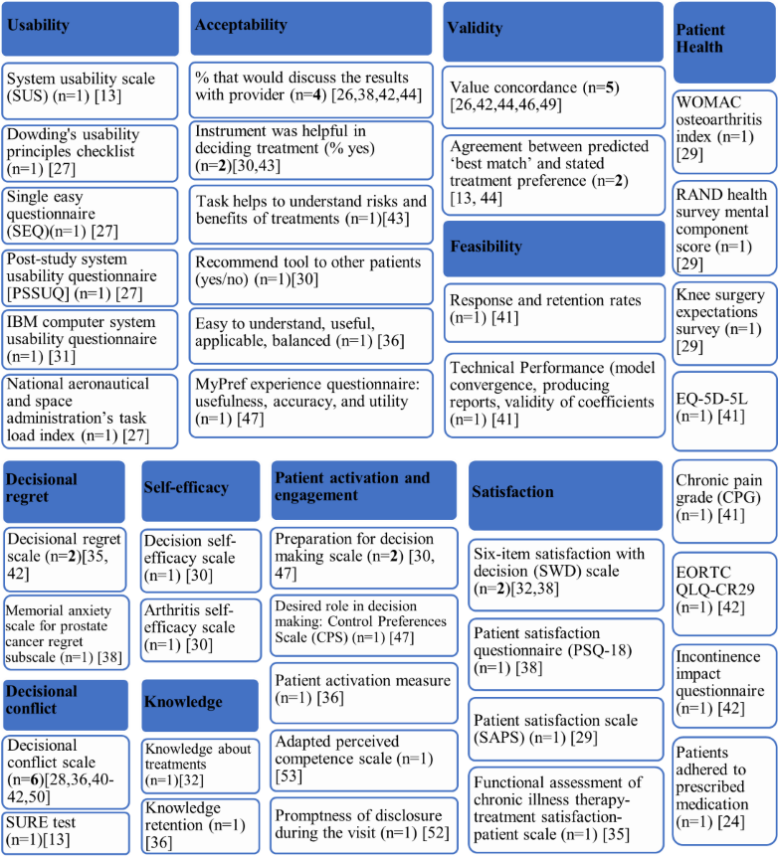
Outcomes. SURE: Sure of myself; Understand information; Risk-benefit ratio; Encouragement.

Figure 10 shows the breadth of outcomes used to evaluate the decision tools, with over 40 different outcomes included. These outcomes fall into 6 core areas including usability, acceptability, validity, feasibility, informed decision making and patient health outcomes. A variety of instruments were used to assess the usability or user-friendliness of the tools, these include the system usability scale, Dowding's usability principles checklist, single easy questionnaire, poststudy system usability questionnaire, IBM computer system usability questionnaire, and NASA (National Aeronautics and Space Administration) task load index (evaluates mental effort needed to perform tasks). While usability was measured using existing validated instruments, accessibility was mostly measured using items developed by the study teams. The most frequently used acceptability item was whether respondents would discuss the results of the tool with their health care professional, followed by how helpful the tool was to patients in deciding which treatment to choose. The validity of the tool was assessed by examining how well its predictions aligned with patient perspectives. Five tools measured “value concordance,” measuring how well the tool's attribute rankings aligned with the patient's own preferences. Two tools measured the similarity between the “best match” treatment generated by the tool and the patient's stated treatment preference. The feasibility of the tool was assessed by recording response rates (n=1) and evaluating the ability of the tool to generate individually tailored reports (n=1).

Informed decision making was assessed using several proxy measures including decisional regret, decisional conflict, self-efficacy, knowledge, patient activation or engagement, and satisfaction. Decisional conflict defined as the “personal uncertainty about which option to choose” was the most commonly used outcome measure (n=6). Decisional regret is defined as “remorse or distress over a decision” and was measured using the 5-item decisional regret scale (n=2) and memorial anxiety scale for prostate cancer regret subscale (n=1). Respondents’ self-efficacy or their confidence to make a treatment decision was measured using the decision self-efficacy scale (n=1) and arthritis self-efficacy scale (n=1). Patients’ active participation and engagement in care were measured using validated instruments including the preparation for decision making scale (n=2), patient activation measure (n=1), control preferences scale (n=1), and adapted perceived competence scale (n=1). Patient satisfaction was also measured using established instruments such as the six-item satisfaction with decision scale (n=2), patient satisfaction questionnaire (n=1), patient satisfaction scale (n=1), and functional assessment of chronic illness therapy-treatment satisfaction-patient scale (n=1). A variety of patient health outcomes were also collected to measure changes to health outcomes after using the tool, further details are available in Figure 10 and Multimedia Appendix 8.

## Discussion

### Principal Findings

With the increasing use of choice experiments embedded in decision tools, this scoping review was conducted to map the evidence base in terms of the methods, design, and evaluation of these tools. This review identified 22 decision tools containing a choice experiment for a variety of health conditions including musculoskeletal conditions, oncological conditions, and chronic conditions. The development of these decision tools was led by the United States with the remaining tools originating in the Netherlands, United Kingdom, Canada, and Australia. A notable trend is the increasing use of choice experiments as a value clarification exercise within decision aids, particularly from 2015 onwards. Variations were observed in how the findings from the choice experiment were presented to patients. Most patients received feedback on attribute importance, while only a minority received a “best match” ranking of treatment options. Diverse approaches to presenting probabilities to participants were identified, with a common approach being the use of mixed methods such as percentages, natural frequencies, and icon arrays. A lack of consensus on the terminology used to describe the developed tools was found. Variation was noted in the study designs and outcome measures used to evaluate the tools. The decisional conflict scale was the most frequently used outcome measure, but no other instrument was widely adopted.

This review also details four relatively new proof-of-concept methods for embedding choice experiments in decision aids, that have been published since 2020. These 4 novel methods include analyzing previous choice experiment data to develop algorithms (scenario analyses) containing expected choice probabilities of treatments according to response patterns [13], classifying respondents into known preference phenotypes [14], using a Bayesian collaborative filtering model [54], or using a penalized multinomial logit model [41]. Developers attempting to embed choice experiments in decision tools are faced with some key challenges: (1) ensuring real-time availability of choice experiment results to patients, (2) tailoring the choice experiment results by incorporating heterogeneity within choice experiment models, and (3) minimizing the number of choice tasks to maintain user engagement alongside the existing information presented in the decision aid. While the new proof-of-concept methods demonstrate potential solutions to these challenges, their validity remains to be conclusively demonstrated. Current evidence is limited to proof-of-concept studies either using small sample sizes or using only simulated data. So, further research is needed to rigorously evaluate these methods and establish their validity.

### Comparison With Previous Work

One of the notable findings of this review in comparison to the one conducted in 2018 is that more developers are using choice experiments as a value clarification method in decision tools. In 2018, Weernink et al [7] only identified 8 studies, but since then a further 14 tools have been developed. Despite this increase in research, many of the characteristics of these tools remain unchanged when compared to the results of the previous review [7]. For example, to present the relative importance of attributes it was common to use bar graphs where longer bars reflect relatively more important attributes. Notably, a protocol for a study exploring alternative visual presentations (ie, bar graph, gauge graph, narrative visualization, and line graph) was included in this review [27]. This represents a positive step towards gathering evidence on how to improve the user experience of these tools. Similarly, limited research exists on how to present the “best matched” treatment. Since presenting balanced information is a key criterion of a good decision tool [58], the table format of the choice experiment should lend itself well to presenting this result. Hazelwood et al [13] demonstrated a potential approach using this format. However, further research is needed to evaluate if this presentation method is understood by patients.

This review highlighted the diverse approaches used by studies to present probabilities to participants. It was common to use mixed methods such as percentages, natural frequencies, and icon arrays to describe risk or efficacy attributes in an accessible way to patients. The most common combination was to use natural frequencies and icon arrays to explain probabilities. This finding is congruent with the review conducted by Trevena et al [59] who also found that using visual formats alongside numerical formats helps to improve the understanding of probabilities. However, when combining different methods to display probabilities, caution should be used as there is also evidence to show that when certain methods are combined, such as verbal and numerical formats, probabilities can be overestimated [60]. Presenting probabilities in an unbiased way is challenging, and there is a vast literature exploring how best to do this [59,60]. Bonner et al [60] recommend using consistent numerical formats, such as “x in 100 over 5 years,” to display probabilities.

Over 40 different outcome measures were used during the evaluation of decision tools. The decisional conflict scale emerged as the most frequently used outcome measure. Besides the decisional conflict scale, no other instrument was widely adopted to evaluate these decision tools. This variability in outcome measurement highlights the lack of consensus on a core set of outcomes to evaluate these tools. These findings were consistent with previous reviews of decision aids documenting inadequate reporting of details of outcome measures [61], which hinders the development of a robust evidence base of the effectiveness of decision aids.

### Limitations

Several limitations should be considered when interpreting the findings of this scoping review. First, the study selection and extraction were performed by a single author. Despite this, the robustness of the selection process was ensured by following the protocol and making selection decisions in conjunction with the wider study team on articles that required a second opinion. Second, the initial search strategy may not have captured all relevant terms used to describe these tools. The included studies used diverse terminology, including “decision-making tool,” “decision aid,” “patient preference elicitation instrument,” and “discussion prioritization tool.” This lack of consistent terminology hindered the identification of potentially relevant studies. To address this potential limitation, an updated search was conducted in September 2023, but it did not yield any additional relevant studies. Although an updated search was conducted using additional terms, it remains possible that some tools were missing. Future reviews could benefit from a more iterative approach to search term development. Third, a risk of bias assessment was not conducted in this review. This approach was selected as aligned with the primary objective of the review, which was to provide a comprehensive overview of the existing literature.

### Future Directions

The findings of this review point to several areas for future research. Further investigation into the newer proof-of-concept methods identified is warranted, as these methods are still in their infancy more research is needed to validate their effectiveness. Moreover, research on the optimal presentation of choice experiment results to patients is also needed. This includes exploring different visual formats and conveying “best match” treatment information in a clear, unbiased, and understandable way. Furthermore, the development of a core set of outcome measures for evaluating decision tools that incorporate choice experiments would be beneficial. This would facilitate comparisons across studies and help build a more robust evidence base regarding the effectiveness of these tools. Achieving consensus on the terminology used to describe these tools would aid in indexing and retrieving relevant literature, facilitating future research. In addition, future studies should provide more detail on the model configurations used to enable real-time personalization, beyond just naming the regression model, to improve transparency. Moreover, to maintain their relevance, these decision aids must be adaptable, requiring ongoing updates to incorporate the latest advancements in treatment options. Further research is needed to determine how these tools can be efficiently updated to reflect the rapidly changing medical field. Finally, integrating decision aids effectively within existing electronic systems in hospitals requires further investigation. Implementation of these tools within existing patient portals or linking them with electronic medical records is likely to increase uptake and facilitate more informed and productive discussions with their health care providers. So future research could explore innovative delivery methods to incorporate these tools into routine care.

### Conclusion

This scoping review provides a comprehensive overview of the different approaches to embedding choice experiments in decision tools. It highlights key considerations for future studies, including the choice of models used, the presentation of information to patients, and the selection of appropriate outcome measures for evaluating the tools. While several challenges remain, the field is rapidly evolving, and the findings of this review provide a foundation for further research and development in this area. The increasing use of digital technologies, including artificial intelligence, offers possibilities to enhance the interactivity, personalization, accessibility, and integration of decision aids into routine care.

## Appendix

Multimedia Appendix 1 Preferred Reporting Items for Systematic reviews and Meta-Analyses extension for Scoping Reviews (PRISMA-ScR) Checklist.

Multimedia Appendix 2 Search Strategy.

Multimedia Appendix 3 List of excluded references.

Multimedia Appendix 4 Further description of attributes and levels.

Multimedia Appendix 5 Further details of the choice tasks.

Multimedia Appendix 6 Design features of the tools.

Multimedia Appendix 7 Evaluation of the decision tool.

Multimedia Appendix 8 Further details of outcomes.

## Abbreviations

COMET: Core Outcome Measures in Effectiveness Trials
DCE: discrete choice experiment
IPDAS: International Patient Decision Aids Standards
pMNL: penalized multinomial logit model
NASA: National Aeronautics and Space Administration
PRISMA: Preferred Reporting Items for Systematic Reviews and Meta-Analyses
PRISMA-ScR: Preferred Reporting Items for Systematic Reviews and Meta-Analyses extension for Scoping Reviews
RCT: randomized controlled trial

## Data Availability

All data generated during this study are included in this published article and Multimedia Appendices 1-8.
