# Supplementary material for: Embedding a Choice Experiment in an Online Decision Aid or Tool: Scoping Review
Source: J Med Internet Res. 2025 Mar 21;27:e59209. doi: 10.2196/59209 (PMC11971581; doi:10.2196/59209)
Supplement: Multimedia Appendix 2 [file jmir_v27i1e59209_app2.docx]

# How to embed a choice experiment in an online decision aid or tool: a scoping review

## Appendix II: Search Strategy

**Search Strategy Database: Ovid MEDLINE(R) and Epub Ahead of Print, In-Process, In-Data-Review & Other Non-Indexed Citations and Daily 1946 to March 09, 2023**

| # | Search Query | Results |
| --- | --- | --- |
| 1 | ("conjoint analysis" or "conjoint measurement" or "conjoint studies" or "conjoint choice experiment" or "discrete choice conjoint experiment" or "discrete choice experiment" or "pairwise choices" or "Best-Worst Scaling" or "Best Worst Scaling" or "MaxDiff Scaling" or "Maximum Difference Scaling" or "ranking conjoint" or "rating conjoint" or "adaptive conjoint analysis" or "adaptive choice based conjoint" or "choice based analysis" or "full profile conjoint" or "choice based conjoint" or "choice set" or "relative preference weight" or "hypothetical scenario" or "stated preference").mp. | 5056 |
| 2 | "decision aid".mp. | 2726 |
| 3 | "individual* utilit*".mp. | 111 |
| 4 | (value* and clarif*).mp. | 26201 |
| 5 | "decision analysis".mp. | 5971 |
| 6 | (individual* adj2 preference).mp. | 1015 |
| 7 | ("shared decision" or "medical decision" or "clinical decision" or "treatment decision").mp. | 74254 |
| 8 | (treatment adj2 choice).mp. | 44791 |
| 9 | (individual adj2 analysis).mp. | 9554 |
| 10 | predict* probabilit*.mp. | 4371 |
| 11 | (decision adj2 tool).mp. | 4725 |
| 12 | or/2-11 | 168645 |
| 13 | 1 and 12 | 563 |
| 14 | ("multi-criteria decision" or "MCDA" or "analytical hierarchy process" or "ahp" or "time trade-off" or "standard gamble" or TTO).mp. | 7864 |
| 15 | 13 not 14 | 513 |
| 16 | limit 15 to english language | 511 |
| 17 | limit 16 to yr="2016 - 2023" | 370 |

**Search Strategy Database: APA PsycInfo 1806 to February Week 4 2023**

| # | Search Query | Results |
| --- | --- | --- |
| 1 | ("conjoint analysis" or "conjoint measurement" or "conjoint studies" or "conjoint choice experiment" or "discrete choice conjoint experiment" or "discrete choice experiment" or "pairwise choices" or "Best-Worst Scaling" or "Best Worst Scaling" or "MaxDiff Scaling" or "Maximum Difference Scaling" or "ranking conjoint" or "rating conjoint" or "adaptive conjoint analysis" or "adaptive choice based conjoint" or "choice based analysis" or "full profile conjoint" or "choice based conjoint" or "choice set" or "relative preference weight" or "hypothetical scenario" or "stated preference").mp. | 3231 |
| 2 | "decision aid".mp. | 916 |
| 3 | "individual* utilit*".mp. | 114 |
| 4 | (value* and clarif*).mp. | 6216 |
| 5 | "decision analysis".mp. | 1039 |
| 6 | (individual* adj2 preference).mp. | 889 |
| 7 | ("shared decision" or "medical decision" or "clinical decision" or "treatment decision").mp. | 11731 |
| 8 | (treatment adj2 choice).mp. | 3399 |
| 9 | (individual adj2 analysis).mp. | 3270 |
| 10 | predict* probabilit*.mp. | 643 |
| 11 | (decision adj2 tool).mp. | 759 |
| 12 | or/2-11 | 28025 |
| 13 | 1 and 12 | 152 |
| 14 | ("multi-criteria decision" or "MCDA" or "analytical hierarchy process" or "ahp" or "time trade-off" or "standard gamble" or TTO).mp. | 1720 |
| 15 | 13 not 14 | 141 |
| 16 | limit 15 to english language | 133 |
| 17 | limit 16 to yr="2016 - 2023" | 62 |

**Search Strategy Database: Web of Science Core Collection (Science Citation Index Expanded (SCI-EXPANDED)-1900-present**

| # | Search Query | Results |
| --- | --- | --- |
| 1 | TS=("conjoint analysis" or "conjoint measurement" or "conjoint studies" or "conjoint choice experiment" or "discrete choice conjoint experiment" or "discrete choice experiment" or "pairwise choices" or "Best-Worst Scaling" or "Best Worst Scaling" or "MaxDiff Scaling" or "Maximum Difference Scaling" or "ranking conjoint" or "rating conjoint" or "adaptive conjoint analysis" or "adaptive choice based conjoint" or "choice based analysis" or "full profile conjoint" or "choice based conjoint" or "choice set" or "relative preference weight" or "hypothetical scenario" or "stated preference") | 10524 |
| 2 | TS=("decision aid") | 4634 |
| 3 | TS=("individual* utilit*") | 460 |
| 4 | TS=(value* and clarif*) | 35770 |
| 5 | TS=("decision analysis") | 16389 |
| 6 | TS=(individual* near/2 preference) | 7288 |
| 7 | TS=("shared decision" or "medical decision" or "clinical decision" or "treatment decision") | 59615 |
| 8 | TS=(treatment near/2 choice) | 40118 |
| 9 | TS=(individual near/2 analysis) | 29771 |
| 10 | TS=(predict* probabilit*) | 124052 |
| 11 | TS=(decision near/2 tool) | 23131 |
| 12 | #11 OR #10 OR #9 OR #8 OR #7 OR #6 OR #5 OR #4 OR #3 OR #2 | 330604 |
| 13 | #12 AND #1 | 1136 |
| 14 | TS=("multi-criteria decision" or "MCDA" or "analytical hierarchy process" or "ahp" or "time trade-off" or "standard gamble" or TTO) | 40626 |
| 15 | (#13) NOT #14 | 1061 |
| 16 | (#13) NOT #14 | 1019 |
| 17 | (#13) NOT #14 and (PY==("2023" OR "2022" OR "2021" OR "2020" OR "2019" OR "2018" OR "2017" OR "2016")) | 677 |
| 18 | (#13) NOT #14 and 2023 or 2022 or 2021 or 2020 or 2019 or 2018 or 2017 or 2016 (Publication Years) and English (Languages) | 675 |
