# Supplementary material for: Embedding a Choice Experiment in an Online Decision Aid or Tool: Scoping Review
Source: J Med Internet Res. 2025 Mar 21;27:e59209. doi: 10.2196/59209 (PMC11971581; doi:10.2196/59209)
Supplement: Multimedia Appendix 3 [file jmir_v27i1e59209_app3.docx]

# How to embed a choice experiment in an online decision aid or tool: a scoping review

## Appendix III: List of excluded references

1. Weernink, M.G., et al., Individual value clarification methods based on conjoint analysis: A systematic review of common practice in task design, statistical analysis, and presentation of results. Medical Decision Making, 2018. 38(6): p. 746-755.

2. Witteman, H.O., et al., Clarifying Values: An Updated and Expanded Systematic Review and Meta-Analysis. Medical Decision Making, 2021. 41(7): p. 801-820.

3. Harrison, M., et al., Do patients and health care providers have discordant preferences about which aspects of treatments matter most? Evidence from a systematic review of discrete choice experiments. BMJ Open, 2017. 7(5): p. e014719.

4. Yelverton, V. and C. Juhnke, Towards personalized healthcare - how can we assess individual patient preferences using discrete choice experiments and best-worst scaling? A systematic literature review. Value in Health, 2020. 23: p. S327-S327.

5. Byrne, M.M., R.J. Thurer, and J.L. Studts, Individual decision making about lung cancer screening: A conjoint analysis of perspectives among a high‐risk national sample. Cancer Medicine, 2019. 8(12): p. 5779-5786.

6. Pignone, M.P., et al., Comparing 3 techniques for eliciting patient values for decision making about prostate-specific antigen screening: a randomized controlled trial. JAMA internal medicine, 2013. 173(5): p. 362-368.

7. Vigneau, C., et al., "Doctor, can I have less frequent injection with highly efficient treatment?" A patient centered study using an electronic choice-based conjoint analysis (ePRO) to assess real world preferences regarding erythropoiesis stimulating agent to treat anaemia in chronic kidney disease (PERCEPOLIS study). Nephrologie et Therapeutique, 2019. 15(3): p. 152-161.

8. Al-Omari, B., J. Farhat, and M. Shraim, The Role of Web-Based Adaptive Choice-Based Conjoint Analysis Technology in Eliciting Patients' Preferences for Osteoarthritis Treatment. International Journal of Environmental Research & Public Health [Electronic Resource], 2023. 20(4): p. 14.

9. Chang, D.H., et al., Incorporating Patient Preferences into a Decision-Making Model of Hand Trauma Reconstruction. International Journal of Environmental Research & Public Health [Electronic Resource], 2021. 18(21): p. 21.

10. Goossens, L.M.A., et al., ABC Index: quantifying experienced burden of COPD in a discrete choice experiment and predicting costs. BMJ Open, 2017. 7(12): p. e017831.

11. Al-Omari, B., et al., Generating Individual Patient Preferences for the Treatment of Osteoarthritis Using Adaptive Choice-Based Conjoint (ACBC) Analysis. Rheumatology & Therapy, 2017. 4(1): p. 167-182.

12. Al-Omari, B. and P. McMeekin, Patients' Preferences Regarding Osteoarthritis Medications: An Adaptive Choice-Based Conjoint Analysis Study. Patient Preference and Adherence, 2020. 14: p. 2501-2515.

13. Ten Broeke, A., et al., BAIT: A New Medical Decision Support Technology Based on Discrete Choice Theory. Medical Decision Making, 2021. 41(5): p. 614-619.

14. Yazdani, S. and M.P. Jadidfard, Developing a decision support system to link health technology assessment (HTA) reports to the health system policies in Iran. Health Policy & Planning, 2017. 32(4): p. 504-515.

15. Kim, J.E., et al., Specificity of clinical decision support tools for vedolizumab and ustekinumab in biologically naive patients with Crohn's disease. Scandinavian Journal of Gastroenterology, 2022. 57(4): p. 446-448.

16. Thomson, M.C., et al., Framing Benefits in Decision Aids: Effects of Varying Contextualizing Statements on Decisions About Sacubitril-Valsartan for Heart Failure. MDM Policy & Practice, 2021. 6(2): p. 23814683211041623.

17. Coxeter, P.D., C.B. Del Mar, and T.C. Hoffmann, Preparing Parents to Make An Informed Choice About Antibiotic Use for Common Acute Respiratory Infections in Children: A Randomised Trial of Brief Decision Aids in a Hypothetical Scenario. The Patient: Patient-Centered Outcomes Research, 2017. 10(4): p. 463-474.

18. Shapiro, L.M., S.L. Eppler, and R.N. Kamal, The Feasibility and Usability of a Ranking Tool to Elicit Patient Preferences for the Treatment of Trigger Finger. Journal of Hand Surgery-American Volume, 2019. 44(6): p. 480-+.

19. Valentine, K., et al., Using standardized videos to examine the validity of the Shared Decision Making Process Scale: Results of a randomized online experiment. Medical Decision Making, 2022. 42(1): p. 105-113.
