# Supplementary material for: Embedding a Choice Experiment in an Online Decision Aid or Tool: Scoping Review
Source: J Med Internet Res. 2025 Mar 21;27:e59209. doi: 10.2196/59209 (PMC11971581; doi:10.2196/59209)
Supplement: Multimedia Appendix 4 [file jmir_v27i1e59209_app4.docx]

# How to embed a choice experiment in an online decision aid or tool: a scoping review

## Appendix IV: Further description of attributes and levels

| Study | No. Of Attributes | Number of Levels | Type of Attributes | Presentation of Probabilities |
| --- | --- | --- | --- | --- |
| (Abraham et al., 2015) | 7 | Five attributes with three levels, two attributes with two levels | Efficacy, side effects, limits on daily activity, administration | Risk presented as natural frequencies (10 out of 100 people will have a heart attack over five years) and using icon arrays of faces |
| (Almario et al., 2018) | 9 | 8 attributes with 4 levels, 1 attribute with 3 levels | Efficacy, side effects, administration, How the medication works | Effectiveness presented as a probability (40% chance my symptoms will improve within 2 months) and side effects as natural frequencies (7 out of 10,000 develop lymphoma) in the choice task. Also, graphics and videos were used to explain risks in the 'learn more' section. |
| (Chhatre et al., 2021) | 11 | All had 3 levels each | Efficacy, side effects, duration, out-of-pocket expenses, caregiver burden, limits on daily activity | Risk presented narratively with percentages in brackets (i.e. more than half (60%)). |
| (Cole et al., 2022) | 5 | All had 3 levels each | Efficacy, time in hospital, side effects | Effectiveness presented as a probability (50% chance of complete remission), and side effects presented narratively (i.e. Mild, moderate). DCE risk info not presented visually. |
| (De Achaval et al., 2012) | 8 | All had 4 levels each | Efficacy, side effects, days in hospital, need for future treatments, limits on daily activity | Risk presented as natural frequencies (1 in 1000 people die during or soon after surgery) and using icon arrays of faces. |
| (Dowsey et al., 2016) | 6 | All had 3 levels each | Efficacy, side effects | Both natural frequencies (79 out of 100 people don't have the risk; 21 out of 100 people have the risk) and icon arrays of people. |
| (Fraenkel et al., 2007) | 5 | Not clearly described | Administration, efficacy, side effects | Natural frequencies (3 out of 10 people have less pain) and pictographs of faces. |
| (Goodsmith et al., 2021) | 4 | All had 2 levels each | Location, type of provider, delivery mode (Internet or in person), mode of intervention (individual or group). | N/a |
| (Hawley et al., 2016) | 4 | All had 2 levels each | Efficacy, appearance, need for additional treatment | No visuals, probabilities (5 % risk of the cancer coming back in 10 years) and words (lLow likelihood (8–10 %) of needing radiation) were used. |
| (Hazlewood et al., 2020) | 8 | 3 attributes with 3 levels, 4 attributes with 2 levels, 1 attribute with 7 levels | Efficacy, side effects, administration, checkups, limits on daily activity | The risk info is presented as natural frequencies (30 out of 100 people) in the DCE task; in the introduction to the DCE, risks were presented as an icon array with faces. |
| (Hess et al., 2015) | 8 | Five attributes with three levels, two attributes with four levels, one attribute with two levels | Efficacy, side effects, treatment location, cost, administration, permanence, recovery time | No details provided |
| (Hutyra et al., 2019) | 4 | All had 3 levels each | Efficacy, recurrence, limits on daily activities, cost | Unclear if icon arrays or natural frequencies were used. |
| (R. Jayadevappa et al., 2019; Ravishankar Jayadevappa et al., 2019) | 15 | All had 3 levels each | Efficacy, side effects, treatment duration, recovery time, administration, cost, impact on social life | Text with percentage in brackets (more than half (60%) may experience urinary function problems in the short term). |
| (Johnson et al., 2016) | Not described | Not described | Efficacy, recovery time | Not described |
| (Loria-Rebolledo et al., 2022) | 8 | All had 2 levels each | Efficacy, side effects, administration | Using words (i.e. side effects - likely) |
| (Pieterse et al., 2019) | 3 | All had 3 levels each | Efficacy, side effects | Rating of attributes task included two risk dots graphs. In the paired comparison task, risks were presented using natural frequencies (i.e. recurrence of the tumour at the site of the 100 people: 10 will, 90 will not.) |
| (Pieterse et al., 2010) | 4 | 3 attributes with 3 levels, 1 attribute with two levels | Efficacy, side effects | Rating of attributes task included two risk dots graphs. Paired comparison task presented probabilities as natural frequencies (40 out of 100 people). |
| (Fraenkel, 2010; Rochon et al., 2014) | 6 | All had 3 levels each | Administration, efficacy, side effects, cost | Used both probabilities and natural frequencies - causes stomach upset in 30% (30 in 100 people) |
| (Snaman et al., 2019; Snaman, et al., 2021) | 9 | Four with 2 levels each and 5 with three levels each | Efficacy; side effects; treatment location; administration; quality of life; survival; frequency of clinic visit; chance of being hospitalized | Numerically: 5% chance of survival |
| (Streufert et al., 2017) | 5 | All had 3 levels each | Efficacy, recurrence, limits on daily activities, cost | Probabilities (80% chance of another shoulder injury) |
| (Byrne et al., 2019; Studts et al., 2020) | 5 | 3 attributes with 3 levels and 2 attributes with 4 levels. | Mortality, false positive, cost, provider recommendation, access to location. | Probabilities (40% false positive rate) |
| (Wittink et al., 2018) | 5 | Not described | Stress, safety, food, money, mobility | N/a |
