# Supplementary material for: Embedding a Choice Experiment in an Online Decision Aid or Tool: Scoping Review
Source: J Med Internet Res. 2025 Mar 21;27:e59209. doi: 10.2196/59209 (PMC11971581; doi:10.2196/59209)
Supplement: Multimedia Appendix 5 [file jmir_v27i1e59209_app5.docx]

# How to embed a choice experiment in an online decision aid or tool: a scoping review

## Appendix V: Further details of the choice tasks

| Study | Type of choice task | Explanation of choice task | No. of choice tasks |
| --- | --- | --- | --- |
| Abraham et al. 2015 | Modified adaptive conjoint analysis | First respondents picked the attribute that is most important to them, second, the respondents rated the importance of each attribute relative to the most important attribute; followed by paired comparisons. | not described |
| Almario et al. 2018 | Adaptive choice-based conjoint | Three profiles side by side comparison. Start with “screener tasks" with “unacceptable” and “must-have” rules. | unknown - the respondents continue the task until internal consistency is achieved |
| Chhatre et al. 2021 | Choice‐based adaptive conjoint analysis | There were two parts to the adaptive design, in part 1, the respondents were shown the attributes and asked to rate the importance of the attribute from not important to extremely important. In part 2, based on responses to part 1, a tailored choice‐set was presented which contained different levels of three attributes, and again respondents were asked to choose which treatment they preferred - options include strongly prefer, somewhat prefer, and no preference. | 9 |
| Cole et al. 2022 | DCE | Two treatment profiles | 10 |
| de Achaval et al. 2012 | Modified adaptive conjoint analysis | First respondents picked the attribute that is most important to them out of a list of 8 attributes; second, the respondents rated the remaining 7 attributes relative to the most important attribute on a scale from 1–10 (“not nearly as important” to “just as important”). This is followed by a paired comparison task that contained two attributes at a time. | not described |
| Dowsey et al. 2016 | Efficient design using Ngene software | Pairwise profiles followed by an opt-out option "where patients were asked if, given the scenarios presented, they would still have the operation or prefer to remain in their current health state." | 6 (two blocks) |
| Fraenkel et al. 2007 | Adaptive conjoint analysis | Rating exercise of attributes, subsequently paired comparisons of treatment profiles | exact number unknown |
| Goodsmith et al. 2021 | Conjoint analysis | Paired comparison (3 attributes out of the 4 were shown at a time) | 16 |
| Hawley et al. 2016 | Conjoint analysis | Paired comparison with options: strongly prefer treatment option 1, neutral, strongly prefer treatment option 2 | 16 |
| Hazelwood et al. 2020 | DCE balanced overlap design, using Sawtooth Software | Two treatment profiles | 6 |
| Hess et al. 2015 | Adaptive conjoint analysis | First, respondents rank their preferences across the list of attributes. Second, “respondents are sequentially presented with the ‘best’ and ‘worst’ scenarios (based on their responses to the ranking exercise) of those attributes, where they are able to rate the choices on an interval scale for levels of importance.” Third, paired comparison tasks which were rated on a 9-point scale. | not described |
| Hutyra et al. 2019 | Adaptive conjoint analysis | Paired comparison where respondents were asked to rank the options on a 9-point Likert-type scale (i.e. Strongly prefer left, indifference, strongly prefer right). "The number of attributes per choice task increased incrementally from 2 to 4 as the ACA exercise progressed". | 8 |
| Jayadevappa et al. 2019 | Adaptive conjoint analysis | First, participants were shown an attribute with two different levels and asked to rate how important was the level difference from "not important," "somewhat important," "very important," or "extremely important". Second, respondents were shown a paired comparison with three attributes and asked to indicate their preferences from: "strongly prefer Treatment A," "somewhat prefer Treatment A," "no preference," "somewhat prefer Treatment B," or "strongly prefer Treatment B." | not described |
| Johnson et al. 2016 | Conjoint analysis | No further details provided | not described |
| Loria-Rebolledo et al. 2022 | Experimental design using Ngene (no further details provided) | Paired comparison task | 12 |
| Pieterse et al. 2019 | Adaptive conjoint analysis | Importance questions of the difference between the best and worst levels for each attribute, followed by paired comparison tasks, the first 5 tasks contained two attributes, the second five contained all three attributes | 10 |
| Pieterse et al. 2010 | Adaptive conjoint analysis | Importance questions of the difference between the best and worst levels for each attribute, followed by paired comparison tasks | 12 |
| Rochon et al. 2014 & Fraenkel et al. 2010 | Modified adaptive conjoint analysis | First respondents picked the attribute that is most important to them out of a list of 6 attributes, second, the respondents rated the importance of each attribute relative to the most important attribute on an 11-point scale; followed by paired comparisons. | 18 |
| Snaman et al. 2019;2021 | Adaptive conjoint analysis | First, participants were shown an attribute with two different levels and asked to rate how important was the level difference from "strongly prefer left," to "strongly prefer right". Second, respondents were shown a paired comparison with three attributes and asked to indicate their preferences from: "strongly prefer left," "indifferent" to "strongly prefer right" | 12 |
| Streufert et al. 2017 | Adaptive conjoint analysis | Importance questions of the difference between the best and worst levels for each attribute, followed by paired comparison tasks ("to make the task easier, 2 attributes were shown in the first 2 pairs. An additional attribute was added for every 2 tasks completed until 5 attributes were shown for each alternative" | 10 |
| Studfts et al. 2020 & Byrne et al. 2019 | Conjoint analysis aiming for "orthogonality and parsimony" | Single profile scenario where respondents are given a 9-point Likert-type ratings scale from ‘‘would definitely not get screened’’ to ‘‘would definitely get screened’’ | 20 |
| Wittnik et al. 2018 | Adaptive best-worst conjoint analysis | Three alternative profiles comparison | not described |
