# Supplementary material for: Embedding a Choice Experiment in an Online Decision Aid or Tool: Scoping Review
Source: J Med Internet Res. 2025 Mar 21;27:e59209. doi: 10.2196/59209 (PMC11971581; doi:10.2196/59209)
Supplement: Multimedia Appendix 6 [file jmir_v27i1e59209_app6.docx]

# How to embed a choice experiment in an online decision aid or tool: a scoping review

## Appendix VI: Design features of the tools

| Study | Received a personalised report? | Patients received feedback on attribute importance | How? | Patients received a ‘best match’ treatment | How? |
| --- | --- | --- | --- | --- | --- |
| Abraham et al. 2015 | Not described | Yes | Importance scores shown as a horizontal bar graph, without exact percentages. Patients were told that longer bars represent 'increasing importance'. | No | N/a |
| Almario et al. 2018 | Yes | Yes | Importance scores shown as a horizontal bar graph, with percentages indicating the importance of each attribute. | No | N/a |
| Chhatre et al. 2021 | Yes | Yes | Importance scores shown as a horizontal bar graph, with percentages indicating the importance of each attribute. Out of the 11 attributes, the respondents were shown the top five most important attributes to each participant. | No | N/a |
| Cole et al. 2022 | Yes | Yes | The pilot is testing 4 visualisation methods: 1. Horizontal bar chart; 2. Using a gauge; 3. Line graph to show a change in attribute importance score over time; 4. Narrative and icon image. | No | N/a |
| de Achaval et al. 2012 | Yes | Yes | Importance scores shown as a horizontal bar graph, without exact percentages. Patients were told that longer bars represent 'increasing importance'. | No | N/a |
| Dowsey et al. 2016 | Not described | Not described | Not described | Not described | N/a |
| Fraenkel et al. 2007 | Yes | Yes | Importance scores shown as a horizontal bar graph, with percentages indicating the importance of each attribute. | Yes | A scale ranging from 0 (worst choice) to 100 (best choice) shows the relative ranking of treatment options |
| Goodsmith et al. 2021 | Not described | Not clear | Not clear | No | N/a |
| Hawley et al. 2016 | Yes | Not described | Not described | Yes | Feedback provided as text: "Based on the responses you just gave, the treatment that seems to be the best fit for you is (mastectomy, mastectomy with reconstruction, lumpectomy with radiation)’’. |
| Hazelwood et al. 2020 | Yes | Not clear | Not clear | Yes | The recommended treatment profile was shown in a DCE format, with the actual attribute levels of the real treatment with a probability which shows the 'best match'. |
| Hess et al. 2015 | Yes | Yes | Importance scores shown as a horizontal bar, with percentages indicating the importance of each attribute. In addition, a guide to interpret the bar graph was provided. | No | N/a |
| Hutyra et al. 2019 | Not clear | Yes | Stated preferences were presented in a graph, further details were not provided. | Not described | N/a |
| Jayadevappa et al. 2019 | Yes | Yes | Importance scores shown as a horizontal bar graph, with percentages indicating the importance of each attribute. Out of the 15 attributes, the respondents are shown the top five most important attributes to each participant. | No | N/a |
| Johnson et al. 2016 | Yes | Not clear | Not clear | Yes | Not clearly described |
| Loria-Rebolledo et al. 2022 | Yes | Yes | Importance scores shown in two vertical bar graphs which are ordered from most important to least important. | No | N/a |
| Pieterse et al. 2019 | Yes | Yes | Importance scores shown in a horizontal graph, with percentages indicating the importance of each attribute. | No | N/a |
| Pieterse et al. 2010 | Not described | Yes | Importance scores shown in a horizontal graph with exact percentages, in addition, the minimum and maximum probability for each attribute is shown alongside the bars. | No | N/a |
| Rochon et al. 2014 | Not described | Yes | Importance scores shown as a horizontal bar graph, without exact percentages. | Yes | A scale ranging from 0 (worst choice) to 100 (best choice) showing the relative ranking of all treatment options. |
| Snaman et al. 2019 | Yes | Yes | Importance scores shown in a horizontal graph, with exact percentages. "Longer bars and higher numbers indicate stronger preferences (out of 100)" | No | N/a |
| Streufert et al. 2017 | Not described | Yes | Importance scores shown in a horizontal graph, with exact percentages. | No | N/a |
| Studfts et al. 2020 & Byrne et al. 2019 | Not clear | Not clear | Not clear | No | N/a |
| Wittnik et al. 2018 | Yes | Yes | Importance scores shown as a horizontal bar graph, without exact percentages. | No | N/a |
