# Supplementary material for: Embedding a Choice Experiment in an Online Decision Aid or Tool: Scoping Review
Source: J Med Internet Res. 2025 Mar 21;27:e59209. doi: 10.2196/59209 (PMC11971581; doi:10.2196/59209)
Supplement: Multimedia Appendix 7 [file jmir_v27i1e59209_app7.docx]

# How to embed a choice experiment in an online decision aid or tool: a scoping review

## Appendix VII: Evaluation of the decision tool

| Study | Evaluation | Sample Size | Mean Duration |
| --- | --- | --- | --- |
| Randomised Control Trial (RCT) | | | |
| Hutyra et al. 2019 | 2 arm RCT - intervention group completed decision tool, control group completed questionnaire about operative and nonoperative treatments | 200 | Not described |
| Hess et al. 2015 | 2 arm RCT - intervention group received the preference elicitation tool and control group received usual counselling at the initial clinic visit, follow-up conducted 6 weeks after the clinic visit | 374 | 15–20 min |
| De Achaval et al. 2012 | 3 arm RCT -1: Adaptive conjoint analysis (ACA) and Video booklet, 2: Video booklet only, 3: standard educational materials. | 208 | Booklet: 20 minutes; Video: 45 minutes; Video + ACA: 60 minutes |
| Jayadevappa et al. 2019 | 2 arm RCT - intervention arm completed the tool and the control arm received standard educational material | 743 | 30 minutes |
| Dowsey et al. 2016 | 2 arm RCT- intervention group complete the decision aid, control group complete a survey, follow-up planned at 12 months. Orthopaedic surgeons will complete a brief expectations survey *(Protocol stage)* | 132 | 30 minutes |
| Pilot RCT | | | |
| Wittnik et al. 2016,2018 | Pilot RCT - intervention group completed the tool and control group received care as usual | 60 | Not described |
| Loria-Rebolledo et al. 2022 | Pilot RCT- intervention group completed the tool and control group got usual care; debrief telephone interviews of intervention group participants to share their experiences of using the UPP DAT; telephone debrief interviews with pharmacists on the potential usefulness of the tool in a clinical setting | 60 | Not described (protocol) |
| Fraenkel et al. 2007 | Pilot RCT study – intervention group completed the computer tool and control group received an information pamphlet | 87 | Not described |
| Hawley et al. 2016 | Pilot RCT study – intervention group viewed the decision aid first; the control group completed a survey before viewing the decision aid | 101 | Not described |
| Mixed methods study | | | |
| Abraham et al. 2015 | Mixed methods study - Qualitative interviews with patients and longitudinal monitoring of treatment adherence | 201 (quant)  56 (qual) | Participants were scheduled for a single 45–60-minute face-to-face interview. Tool completion time not provided. |
| Snaman et al. 2021 | Mixed methods pilot; semi-structured interviews and questionnaires 1-2 weeks after completing the survey. Investigators used a ‘weaving approach’ (Fetters et al., 2013) to combine quantitative and qualitative results. | 34 (15 patients, 7 parents, 12 HCP) |  |
| Goodsmith et al. 2021 | Mixed methods study - questionnaire and semi-structured interviews | 35 (quant),  23 (qual) | 6.6 minutes |
| Cole et al. 2022 | Mixed methods study, qualitative interviews and quantitative surveys of patients and HCPs (Protocol stage) | Approximately 75 | N/a |
| Cohort study | | | |
| Chhatre et al. 2021 | Prospective cohort study (baseline and after tool) | 108 | 30 mins |
| Johnson et al. 2016 | Cohort study (before and after tool completion) | 109 | Not described |
| Studfts et al. 2020 ; Byrne et al. 2019 | Cohort study (pre-post design) | 210 | Not described |
| Pieterse et al. 2019 | Prospective cohort study- the intervention was offered to a subset of consecutive patients before the pre-treatment consultation at multiple centres. Collected long-term follow-up data at 6 months and 12 months. | 135 | Not described |
| Cross-sectional study | | | |
| Hazelwood et al. 2020 | Cross-sectional study: post tool evaluation of outcomes. | 29 | 15 minutes |
| Streufert et al. 2017 | Survey | 501 | 13 minutes 37 seconds |
| Jayadevappa et al. 2009a | Survey after completing the tool | 52 | 30 minutes |
| Other | | | |
| Pieterse et al. 2009 | Non-randomised comparison of computer-aided personal interview (CAPI) group vs the internet group | 115 | Approximately 16 minutes |
| Rochon et al. 2014 | Qualitative study: Focus groups | 5 groups, 29 respondents | Not described |
| Almario et al. 2018 | Did not conduct a formal evaluation | N/a | N/a |
