# Supplementary material for: Embedding a Choice Experiment in an Online Decision Aid or Tool: Scoping Review
Source: J Med Internet Res. 2025 Mar 21;27:e59209. doi: 10.2196/59209 (PMC11971581; doi:10.2196/59209)
Supplement: Multimedia Appendix 8 [file jmir_v27i1e59209_app8.docx]

# How to embed a choice experiment in an online decision aid or tool: a scoping review

## Appendix VIII: Further details of outcomes

| Study | Outcome |
| --- | --- |
| Abraham et al. 2015 | Adherence: persistence of drug use as a surrogate measure for adherence, measured over a year by examining patient prescriptions to see if patients adhered to the prescribed medication. Qualitative interviews on the patients’ experience of completing the preference-elicitation exercise |
| Almario et al. 2018 | N/a |
| Chhatre et al. 2021 | Usability (easy to understand) and acceptability (% that would discuss the results with a provider). Association between treatment uptake and predicted attribute importance |
| Cole et al. 2022 | Usability: Dowding's Usability Principles checklist; Single Easy Questionnaire (SEQ); Post-Study System Usability Questionnaire [PSSUQ]; Cognitive workload: National Aeronautical and Space Administration’s Task Load Index [NASA-TLX]; performance: time taken for task completion and the number of errors committed |
| de Achaval et al. 2012 | Decision conflict scale (DCS) |
| Dowsey et al. 2016 | Changes in patient reported knee pain and disability between baseline and 12 months post-surgery: WOMAC Osteoarthritis Index; patient satisfaction: Self-administered Patient Satisfaction Scale (SAPS); changes in psychological wellbeing between baseline and 12 months post-surgery: RAND Health Survey mental component score (VR-12); differences in expectations of recovery between baseline and 1 week prior to scheduled surgery: Knee Surgery Expectations Survey; concordance between matched surgeon and patient expectation for recovery following surgery: Knee Surgery Expectations Survey. |
| Fraenkel et al. 2007 | Self-confidence: Decision Self-Efficacy Scale; Perceived usefulness of the intervention: 11-item Preparation for Decision-Making Scale; Self-efficacy: Arthritis Self-Efficacy scale; Acceptability: "rate level of difficulty of the task (4-point scale ranging from “very easy to do” to “very hard to do”), whether or not they would recommend the ACA task for other patients with knee pain (yes/no), and whether or not they felt the ACA bar graph reflected their values (4-point scale ranging from “very much” to “not at all”)." |
| Goodsmith et al. 2021 | IBM computer system Usability Questionnaire (CSUQ) and semi structured interviews asking about user experience. |
| Hawley et al. 2016 | Knowledge about surgical treatments: questions developed by the investigators; decision satisfaction: adapted version of the decision satisfaction scale; perceived values concordance was assessed by asking, ‘‘the surgical treatment decision matches my values’’ |
| Hazelwood et al. 2020 | Value concordance: "percent agreement between the patient’s final treatment choice versus the predicted choice”; decisional conflict scale: SURE (Sure of myself; Understand information; Risk-benefit ratio; Encouragement); user friendliness measure: System usability Scale (SUS) |
| Hess et al. 2015 | Decision Regret Scale; The Functional Assessment of Chronic Illness Therapy-Treatment Satisfaction-Patient Scale |
| Hutyra et al. 2019 | Respondents preferred choices were assessed for alignment with evidence; decisional conflict scale (DCS); Patient Activation Measure; knowledge retention; acceptability: adapted questions from an osteoporosis-based decision tool |
| Jayadevappa et al. 2019 | Pilot cross sectional study assessed feasibility and acceptability using questions developed by the study team. RCT: patient satisfaction with care: patient satisfaction questionnaire (PSQ-18); satisfaction with decision: six-item satisfaction with decision (SWD) scale; decision regret: the memorial anxiety scale for prostate cancer (MAX-PC); and alignment of treatment choice according to cancer risk. |
| Johnson et al. 2016 | Decisional conflict scale |
| Loria-Rebolledo et al. 2022 | Technical performance of the tool: model convergence, producing individual reports, and the face validity of parameter estimates; Feasibility of collecting data: recruitment Processes, Response and Retention Rates, Timeline and Resource Requirements; Secondary outcomes: Personal Well-Being scale, EQ-5D-5L, Chronic Pain Grade (CPG) and Decisional Conflict Scale, |
| Pieterse et al. 2019 | Decisional Conflict: Decisional Conflict Scale; decision regret: five-item Decisional Regret Scale; clinical outcomes: EORTC QLQ-CR29, Incontinence Impact Questionnaire. Qualitative interviews with the intervention group(n=35): “the VCM had provided them insight in treatment benefits and harms, they had printed the results, they had discussed these with the radiation oncologist, and if the VCM had helped them in making the decision”; Values clarity/expression of preferences: coding of audiotapes of the consultation |
| Pieterse et al. 2010 | To evaluate the tool the investigators developed their own questionnaire, questions were about the acceptability of the tool “i.e. I would discuss the results with my specialist; I gained insight in benefits and side effects of treatment from completing the questionnaire; The questionnaire would be helpful in deciding about treatment; Feedback on relative outcome importance was clear” |
| Rochon et al. 2014& Fraenkel et al. 2010 | Interview topic guide included the following: "What was the computer program like for you?’ Additional prompts asked about participants’ understanding and comfort level with the program. Follow-up questions asked whether the program helped patients decide what was important in making a treatment decision with prompts about whether the program reflected their preferences, what they learned from the program and what made it difficult to choose between scenarios. In addition, participants were asked to discuss whether they agreed or disagreed with the preferred treatment predicted by the software." |
| Snaman et al. 2021 | Participants desired role in decision making: Control Preferences Scale (CPS). They also ranked the three most important treatment-related factors, before completing the task. This ranking was used to compare how accurate the predicted rankings from the MYPREF tool. MYPREF Experience Questionnaire (MPEQ), which was developed for the study and focused on the usefulness, accuracy, ease of understanding and utility of the MYPREF tool;10-item preparation for Decision Making (PREPDM) scale. |
| Streufert et al. 2017 | Agreement With Generated Results: they developed a single question on a 5-point Likert-type scale from “very well” to “very poorly.”; willingness to share relative importance scores with clinicians: a single question asking respondents whether they would be willing to disclose the preference weights to their physician. |
| Studfts et al. 2020 & Byrne et al. 2019 | Low literacy version of the Decisional Conflict Scale (DCS) before and immediately after completing the tool |
| Wittnik et al. 2016, 2018 | Stressor disclosure: audio-recording of visit, patient confidence of stressor disclosure: adapted perceived competence scale, patient active participation: audio-recording of visit; promptness of disclosure during the visit: audio-recording of visit |
